# Response to: Plastic Surgery Tourism: Complications, Costs, and Unnecessary Spending?

**DOI:** 10.1093/asjof/ojae102

**Published:** 2024-11-06

**Authors:** Miguel Ángel Rivera Mendoza, Maria Fernanda Santoyo-Barco

We read with great interest the article titled “Plastic Surgery Tourism: Complications, Costs, and Unnecessary Spending?” authored by Hery et al.^1^ This article is highly relevant to contemporary medical literature and reflects the current societal context, where decision making in plastic surgery is often influenced by social media advertizing. We also congratulate the authors for conducting this study despite the limitations in data collection that were mentioned.

The article aims to examine postoperative complications associated with medical tourism in plastic surgery, with seromas and infections being the most significant. We believe that some possible causes may be the low implementation of checklists and clinical practice guidelines, such as the ERAS guidelines,^2^ its performance in locations that do not meet quality standards, and by unqualified personnel, which in turn promotes the false perception of lower costs as demonstrated in the article. However, we consider that these factors are linked to an increase in complications, a lack of satisfaction, and the high cost of managing complications. Moreover, we note that the most popular countries for medical tourism are middle- and high-income countries, such as Mexico, the Dominican Republic, and Turkey,^3^ supporting the lack of rigorous implementation of management guidelines.

Despite the authors’ conclusions, the observed rise in the number of patients pursuing medical tourism challenges the notion that this practice is inherently dangerous. On the contrary, it presents an opportunity to promote high-quality medical care in middle-income countries. For example, in Colombia, aside from the existing legal requirements for practicing plastic surgery, as established by Law 2, Article 214 of 1962, a draft bill was recently introduced to regulate the practice of cosmetic and reconstructive surgery.^4^

We encourage the authors to continue collecting data from more patients to develop strategies to mitigate major complications internationally.

This letter to the editor aims to contribute to the medical literature about medical tourism, the advantages and disadvantages, and a proposal to promote high-quality standards in medical practice worldwide.
